# Synthesized nano particles of glimepiride via spray freezing into cryogenic liquid: characterization, antidiabetic activity, and bioavailability

**DOI:** 10.1080/10717544.2021.2018524

**Published:** 2022-01-24

**Authors:** Dalia A. Gaber, Abdulrahman S. Alhuwaymili, Hessah S. Alhawas, Alhnouf A. Almutiri, Amal M. Alsubaiyel, Siham A. Abdoun, Reem A. Almutairi

**Affiliations:** a Department of Pharmaceutics, College of Pharmacy, AL-Qassim University, Buraidah, Kingdom of Saudi Arabia; b Department of Quality Control & Quality Assurance, Holding Company for Biological Products and Vaccines, Cairo, Egypt; c Novo Nordisk Saudi Arabia, Health Care Riyadh HQ, Riyadh, Kingdom of Saudi Arabia; d College of Pharmacy, AL-Qassim University, Buraidah, Kingdom of Saudi Arabia; e College of Pharmacy, AL-Qassim University, Unaizah, Kingdom of Saudi Arabia

**Keywords:** Antidiabetic, nano size, cryogenic, Glimepiride, bioavailability

## Abstract

The aim of this work was to formulate glimepiride (class II drug) which is characterized by low solubility and high permeability as nanostructured particles using a cryogenic technique with an aid of water-soluble polymer to improve its aqueous solubility and hence its bioavailability. 27 formula of glimepiride nano size particles were prepared by a spray freezing into cryogenic liquid (SCFL) using poly vinyl pyrrolidone K-30 (PVP K-30); that three drug polymer ratio (1:1, 1:2, and 1:3), with three different volumes of feeding solution (50, 100, 150 mL), at three flow rates (10, 20, and 30 mL/min). The prepared formulations were evaluated for production yield, particle size, zeta potential, drug content, release rate, *in vivo* hypoglycemic activity, and bioavailability. All prepared formulations showed high production yield and drug content ranged between 91.1 ± 3.4% and 94.3 ± 1.8% and 95.1 ± 2.8% and 97.1 ± 2.5%, respectively. The mean particles size was ranged between 280 ± 62 nm and 520 ± 30 nm. The results of *in vitro* release study revealed significant enhancement in the solubility of prepared formulations compared with the pure drug. It was found that optimal formula showed a significant reduction in blood glucose levels in diabetic rats, and 1.79-fold enhancements in oral bioavailability compared with market tablets. Nanoparticle prepared by SCFL method is an encouraging formula for improving the solubility and the bioavailability of glimepiride.

## Introduction

**Figure 1.**
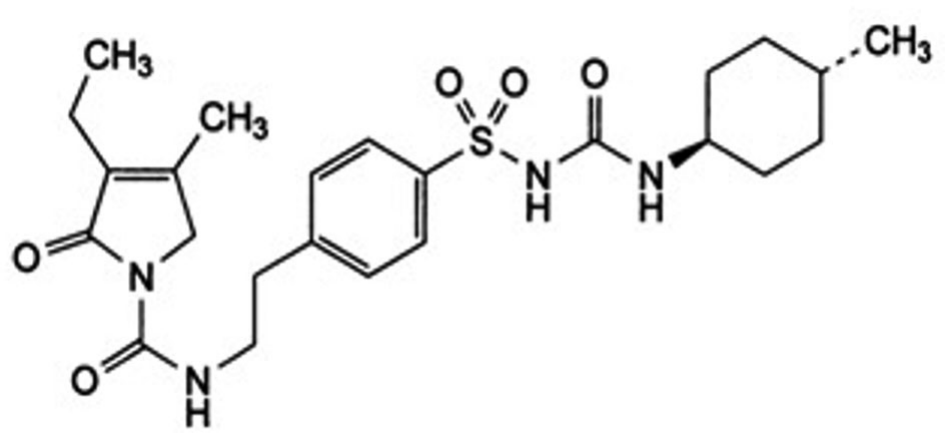
Chemical structure of glimepiride.

Adult-onset diabetes or what is known as Type II diabetes mellitus accounts for more than 90% of all diabetes cases (American Diabetes Association, 2014). Individuals who are diagnosed as diabetics with this type are having insulin deficiency accompanied usually by peripheral insulin resistance (Inzucchi et al., 2012). Insulin is not the optimal choice for them at least initially (Simpson et al., 2006). Definitely, diabetes needs meticulous and continuous medical care for controlling blood glycemic levels within the optimal levels (Tanabe et al., 2017). Oral hypoglycemic drugs are the first-line treatment for more than 85% of diabetic patients, so great concern was paid for their selection, design, and development (Association, 2018). Glimepiride (GP) belongs to sulfonylurea oral antidiabetic drugs (third-generation) that is used in the treatment of type II diabetes mellitus -Figure 1-(Wagh et al., 2012). GP can be considered as one of the most important drugs in this class due to its high hypoglycemic activity, good protein binding, low systemic toxicity, and its possible concomitant use with insulin (González-Ortiz et al., 2009; Gill et al., 2010). GP works by stimulating pancreatic cells (ß-cells) to produce insulin. Biopharmaceutical Classification System classified GP as a class II drug, due to its low aqueous solubility and high permeability characteristics (Gill et al., 2010). Difficulties are facing oral dosage forms of GP due to its low water solubility, poor profiles, and hence low bioavailability (Ning et al., 2011; Vidyadhara et al., 2011). The strategies done to improve the rate and extent of absorption of such drugs are based on enhancing its dissolution profiles in the gastric fluids (Sharma et al., 2019). Many techniques were reported to improve the solubility of poorly soluble drugs; including solid dispersion, co-solvency, salt formation, spray congealing, micro emulsification, electrospray, and cryogenic technology (Williams et al., 2013). Cryogenic spray techniques (CST) are novel efficient methods for size reduction that can be used to improve the dissolution rate of insoluble drugs by generating nanostructured, amorphous, and highly porous particles at low temperatures (Costantino et al., 2000). The cryogenic spray techniques include several types like spray freezing onto cryogenic fluids, spray freezing into vapor over liquid, spray freezing into cryogenic liquids (SFCL), and ultra-rapid freezing to form micro/nano size drug particles with improved wettability. SFCL is a novel, simple, and productive cryogenic process (Liu et al., 2018). It is based on atomizing, an organic or aqueous solution, emulsion, or suspension of the drug and excipients into a compressed liquid (like compressed carbon dioxide liquid, ethane, propane) or the cryogenic liquids (such as argon, nitrogen, or hydrofluoroethers) (Parhizkar et al., 2017). The atomization of the feed mixture into one of the cryogenic liquids produces frozen nanostructured particles which, upon lyophilization, give free-flowing dry nano-size powders (Nath et al., 2013). Rogers et al. (2003) have been reported that the dissolution rate of danazol (poor soluble drug) prepared by SCFL was superior compared with conventional co-grinding and slow freezing size reduction methods. So, the objective of this study was to prepare nanoparticles of GP to increase its solubility and hence its oral bioavailability. The formulations of GP nanoparticles were prepared by the SCFL method with the aid of water soluble polymer. In addition, the influence of the drug-polymer ratio, the volume of the feeding solution, and the feeding rate on the physicochemical properties of formed GP nano size (GPN) powders were studied. The GPN powders prepared using different predetermined processing factors were assessed for the yield, particle size, zeta potential, drug content, and *in vitro* release rate study to selected formulations. Finally, the antidiabetic effect of optimized formula was assessed and its *in vivo* pharmacokinetic parameters were measured in rats.

## Materials and methods

### Materials

GP was a kind gift from Delta for Pharmaceutical Industries, Egypt. Polyvinylpyrrolidone K-30 (PVP K-30) (El Kahera Pharmaceuticals, Cairo, Egypt), poloxamer 188 (Brunsbüttel, Germany). Dimethylfomamide, butanol, and acetonitrile were HPLC grade. Other chemicals were of analytical grade and were used as obtained. Purified water was obtained from an ultra-pure water system (Milli-QUV plus, Millipore S.A., Molsheim Cedex, France).

### Methods

#### Preparation of GP nano sized powders

The feed solution was prepared as follows; GP and PVP K30 in ratios 1:1, 1:2, and 1:3 were dissolved in either 50, 100, or 150 mL acetonitrile, poloxamer 188 (0.05%) was used as a surfactant. The feed solution was pumped at constant pressure 4000 psi from a syringe pump (Model no. 100 DX, ISCO Inc., Lincoln, NE) to provide a flow rate of either 10, 20, or 30 mL/min (Table 1). The feed solution cell was attached to a nozzle (65 µm inner diameter), which was atomized the solution beneath the surface of liquid nitrogen (the cryogenic liquid) (Hu et al., 2003). Frozen particles formed promptly and were collected and dried by lyophilization using Tray Lyophilizer (The VirTis Company, Inc. Gardiner, NY). Lyophilized preparations were kept in a desiccator until further investigation.

**Table 1.** Composition and characterization of GPNs formulations: yield percentage, drug content percentage, particle size, and zeta potential.

Formulation code	GP:PVP K30 ratio	Volume of acetonitrile (mL)	Flow rate mL/min	Percentage of yield	Percentage of drug content	PZ(nm)	ZP(−ve mV)
GPN1	1:1	50	10	93.5 ± 2.1	96.2 ± 1.3	420 ± 35	27 ± 1.2
GPN 2		100		91.6 ± 3.5	95.6 ± 2.6	360 ± 30	25 ± 0.9
GPN 3		150		92.1 ± 3.4	97.1 ± 2.5	325 ± 44	26 ± 1.2
GPN 4		50	20	93.0 ± 2.3	96.0 ± 3.3	405 ± 54	25 ± 2.3
GPN 5		100		91.4 ± 1.3	95.7 ± 2.5	354 ± 59	24 ± 1.7
GPN 6		150		94.2 ± 2.6	96.1 ± 1.3	330 ± 55	20 ± 2.1
GPN 7		50	30	93.1 ± 2.0	96.1 ± 2.5	310 ± 45	27 ± 2.2
GPN 8		100		92.2 ± 3.4	95.6 ± 1.4	280 ± 62	26 ± 1.4
GPN 9		150		91.1 ± 3.4	96.1 ± 2.4	284 ± 54	25 ± 1.8
GPN10	1:2	50	10	94.4 ± 3.1	96.2 ± 1.7	462 ± 25	26 ± 2.4
GPN11		100		92.5 ± 1.5	97.1 ± 2.3	390 ± 26	23 ± 3.1
GPN12		150		94.1 ± 0.8	96.1 ± 1.5	368 ± 31	24 ± 1.2
GPN13		50	20	91.1 ± 3.4	96.2 ± 1.6	380 ± 44	22 ± 2.3
GPN14		100		94.0 ± 1.4	97.0 ± 2.9	361 ± 38	21 ± 1.8
GPN 15		150		92.2 ± 2.5	96.1 ± 1.8	340 ± 41	25 ± 1.9
GPN16		50	30	92.2 ± 3.1	97.1 ± 2.4	325 ± 25	26 ± 2.5
GPN17		100		94.3 ± 1.8	96.8 ± 1.8	306 ± 41	25 ± 2.3
GPN18		150		93.1 ± 2.5	97.0 ± 2.2	291 ± 38	24 ± 2.6
GPN19	1.:3	50	10	92.6 ± 3.1	95.1 ± 2.8	526 ± 30	22 ± 3.2
GPN20		100		90.2 ± 4.5	96.1 ± 1.1	485 ± 37	22 ± 1.5
GPN 21		150		91.3 ± 2.4	95.5 ± 1.1	371 ± 21	25 ± 3.2
GPN22		50	20	91.5 ± 3.2	94.6 ± 2.7	440 ± 44	25 ± 2.4
GPN23		100		93.2 ± 2.3	96.0 ± 2.2	375 ± 28	20 ± 2.5
GPN24		150		93.7 ± 3.6	95.1 ± 3.1	352 ± 30	22 ± 3.4
GPN25		50	30	91.8 ± 1.5	95.5 ± 3.2	328 ± 15	25 ± 1.4
GPN26		100		94.2 ± 4.7	96.4 ± 3.2	318 ± 22	23 ± 2.2
GPN27		150		92.1 ± 0.8	95.4 ± 2.1	310 ± 25	26 ± 3.1

#### Characterization of GP nano sized formulations (GPNs)

##### The production yield of GPN formulations

The production yield of all prepared formulations of P was calculated as the following equation (Sahoo et al., 2017):

(1) $$\text{The production yield\%}=\;\frac{\text{Weight of the collected nanosize particles}}{\text{Total weight of drug and carrier used}}\;\times 100$$

#### Determination of GP content in GPNs formulations

The content of GP in nanosize formulations was determined by RP- HPLC method published by Mohd et al. (2014). Briefly; a Reversed-phase C18 column (250 × 4.6 mm) with particle size 5 µm was used for separation. Acetonitrile and 0.2 M phosphate buffer (pH = 7.4) in a ratio of 40:60 v/v was used as a mobile phase at a flow rate of 1 mL/min, and quantification was achieved with a UV detector at 232 nm. The drug content was calculated based on the amount of drug in the GPNs formula and the amount of GP used for the preparation (Mohd et al., 2014).

#### Particle size and zeta potential analysis

Defining the particle size and zeta potential of GPNs was done using dynamic laser light scattering (Zetasizer Ver. 5.11 Malvern). All GPNs formulations were suspended in deionized water (50 µg/mL) at pH 7 for measurement (Sahoo et al., 2017).

#### Powder X-ray diffraction test (XRDT)

The XRDT was conducted using Copper Potassium alpha 1 radiation at wavelength 1.54054 Å using an X-ray diffractometer (Philip Analytical Inc., Natick, MA). The GPNs powders were placed in a glass sample holder. Samples were scanned from 10° to 50° at a rate of 0.05°/s. Bulk GP powder was used for comparison purposes (Cullity, 2001).

#### Differential scanning calorimetry

The Thermal behavior of GP and selected GPN formulations were deliberated using differential scanning calorimetry technique (DSC) (Mettler Toledo, SwitzerGPNd). 5-mg samples were heated in aluminum pans, at a rate of 10 °C/min at a range 40–200 °C under a nitrogen flow of 50 mL/min.

#### Karl–Fisher analysis

Any residual water in the GPNs powders was measured by a Karl–Fisher Titrator (KFT) (Photovolt Instrument, St. Louis Park, MN). Aliquots of 5 mg samples were tested in the KFT vessel. Each sample was measured in replicates of three (*n* = 3) (Martin, 1993).

#### Gas chromatography analysis of organic solvent residual

The residual organic solvent in the GPNs powders was identified using a gas chromatograph (Hewlett-Packard 5890 A). 5-mg samples were dissolved in dimethylformamide and butanol was used as an internal standard. The calibration standard ranged from 20 to 50,000 ppm for acetonitrile. The calibration standard was used to calibrate the residual organic solvent level in the GPNs powders produced from acetonitrile (Wittaya-Areekul & Nail, 1998).

#### Contact angle measurement

Compacts of sample powders were prepared at a high compression force using 6 mm diameter punches with a flat face (Laboratory Press, Round Rock, TX). A droplet of purified water (5 µL) was placed onto the surface of the compact and observed. The contact angle was measured using contact angle meter Drop Shape Analyzer – DSA100 with ADVANCE software (KRÜSS GmbH Co., Hamburg, Germany) (Binbin et al., 2020).

#### Examination of morphology of the surface of bulk GP and GNP using scanning electron microscope (SEM) technique

The sample of bulk GP powder and an optimized formula of GNPs was uniformLy coated with gold under vacuum and its surface morphology was examined using SEM (Metler Toledo, Tokyo, Japan).

#### Saturation solubility study

The saturation solubility of GPNs formulation in purified water was determined briefly; the excess amount of prepared formulations were added to 5 mL distilled water into centrifugation tubes and centrifuged at 20,000 rpm for 1 h. The supernatant was filtered through 0.2 μm filters and GP content was assessed using RP-HPLC method. Also, the saturation solubility of pure GP in distilled water was determined for comparison purposes (Rahim et al., 2019).

#### *In vitro* dissolution studies

Based on the yield, drug content, and saturation solubility results, three formulations were selected for further *in vitro* release assessment. The amounts of GP released from the selected formulations as a function of time were determined using USP type II apparatus. GPN formulations (10 mg) were packed in a cellophane membrane and added to 900 mL of deionized water at 37 °C. The paddle speed was adjusted at 50 rpm. Samples (5 mL) were taken at each time point, filtered through a 0.45 µm filter, and assayed for GP content using a UV spectrophotometer at 235 nm. Equivalent volumes of fresh medium were added to the dissolution medium to keep it in a constant volume. Each sample was tested triplicate (*n* = 3) (Rakesh & Madhabhai, 2008).

### Statistical analysis

The collected data were compared using a Student’s *t*-test of the two samples supposing equal variances to evaluate the differences. The level of significance (*α* = 0.05) was based on the 95% probability value (*p < .*05).

### Evaluation of the antidiabetic activity of GP from the selected formulations

Eighteen male Wister rats with a weight range betwwen150 and 180 g were used for the evaluation of the antidiabetic effect of GP from GPNs. Rats were divided into three groups each group composed of six rats. All groups were housed and kept under standard laboratory conditions, at the normal room temperature. All experimental protocols were approved by the Research Ethics committee (Number PI/1201). All rats were injected intraperitoneally with 50 mg⁄kg streptozotocin (STZ). After 72 h, the blood glucose level was measured for all rats. Rats that showed blood glucose levels above 250 mg/dL were selected to complete the *in vivo* study (Reginald-Opara et al., 2015). The diabetic rats were divided into three groups (six rats in each group), as follows:

Group I: treated with normal saline (orally) used as a control group for the study.

Group II: treated with pure GP (0.1 mg/kg; orally).

Group III: treated with the selected formula (GPN18) (0.1 mg/kg; orally). After receiving the treatments, blood samples were withdrawn from the tail vein each hour for 8 h. Blood samples were analyzed for the glucose level using a commercial glucose kit.

### *In vivo* pharmacokinetic study of the optimized formula in Wistar rats

The test protocol was performed in agreement with the reported principles of animal care published by The European center for the validation of alternative methods (Diehl et al., 2001). *In vivo* pharmacokinetic study was conducted on eighteen male Wistar rats weighing 200–230 g. Animals were divided into three groups (six rats per group) with unlimited access to water and food before and during the experiment. The first group was received a pre-weighted amount of optimized formula of GPN18 suspended in distilled water (0.1 mg GP/kg suspended in 2 mL water), via oral gavage. Tablets of the marketed product (Glimadel^®^,1 mg) were cursed and the weight of tablets with an equivalent amount of GP was suspended in distilled water and given to the second group. Normal saline was given to rats in the third group which was used as a control group. Blood samples (0.5 mL) were withdrawn in heparinized tubes from the tail vein at 0.5, 1, 2, 3, 4, 6, 8, and 12 h after dosing. The collected blood samples were centrifuged at 4000 RPM for 10 min and were stored at −20 °C until further analysis. The GP concentrations in plasma samples were assayed according to Abdul Bari Mohd method. The method was validated for precision, selectivity, and accuracy prior to the start of the study (Mohd et al., 2014).

### Assessment of GP concentration in plasma and statistical analysis

All pharmacokinetic parameters were deliberate from the plasma concentrations time curve. GP plasma concentrations are presented as the mean ± SD. AUC, *C*_max_, and *t*_max_ were stated as measured.

The relative bioavailability (*F*) with the commercial product was calculated using the following equation:

(2) $$F=\;\frac{\mathrm{AUCtest}}{\mathrm{AUCref}}\times 100$$

### Statistical estimation of the results

All data were evaluated for statistical differences by SPSS Statistics 17 program (Armonk, NY, USA), *p*-value <.05 was considered significant.

## Result and discussion

In the study, 27 formulations of GPNs were prepared through the SFCL process which could be termed as a simple, rapid, and cost-effective technique. Certain process variables were evaluated for their effect on the dissolution rate, antidiabetic activity, and pharmacokinetic profile of GPNs.

### The production yield percentage of GPNs

All GPNs particles were effectively prepared by a cryogenic technique under liquefied nitrogen gas with an accepted production yield percentage ranging between 91.1 ± 3.4% and 94.2 ± 4.7%, as shown in Table 1.

### The drug content percentage drug in GNPs

Data represented in Table 1 showed that; the drug content for all GPNs formulation was ranged between 95.1 ± 2.8% and 97.1 ± 2.5%. The high percentage of drug content showed that the production of GPNs under the selected process conditions is effective and reproducible. These results were in good agreement with the result reported by Hu et al. (2003), who reported that the drug content percentage of carbamazepine in nano-particles prepared by spray freezing liquid technology was exceeding 90.25 ± 1.5%.

### The particle size (PZ) and the zeta potential (ZP) determination

The particle size (PZ) and the zeta potential (ZP) of GPNs particles were measured directly after preparation. The mean PZ and ZP of GPNs are shown in Table 1. GPNs particles size was ranged between 280 ± 62 nm and 520 ± 30 nm for GPN 8 and GPN 19, respectively. GPN 8 was the smallest particle with particle size 280 ± 62 nm and ZP 26 ± 1.4. The results showed that there was an inverse relationship between polymer concentration and particle size, which could be interpreted on the basis of mixture viscosity. The increase in the polymer concentration will increase the viscosity of the drug/polymer mixture which produces bigger droplets upon spraying from the nozzle. The zeta potential was used to measure the surface charge of GPN particles which states the stability of particles in the formulation. The agglomeration of the particles was minimized by the adsorption of polymers which stabilize the particles and afford sufficient repulsion forces between the particles. But our results showed that; a higher concentration of PVP-K30 showed a non-significant increase in the repulsion force at the level *p* ˂ .05, which was in difference with Rabinow (2004), who reported that the higher polymer concentration leads to more repulsion forces between the nanoparticles. This difference in the observations can be interpreted on the basis of using different polymers and due to the differences in experiments conditions. Furthermore, the volume of the solvent (acetonitrile) showed a significant effect on particles growth, results revealed that larger volume helped in stabilizing drug suspension and decreasing its energy (Kamalakkannan et al., 2013). It was reported that solvents in larger volumes were able to decrease the surface free energy by decreasing the agglomeration of particles and improving the adsorption of the polymer over the surface of the particles. Our study observed a reduction in the size of particles as solvent volume increased from 50 to 100 mL but further increase in volume to 150 mL showed a non-significant further decrease in the particle size which may be due to the smallest size has been achieved at 100 mL and no more reduction in size could be achieved under our experimental conditions. An inverse relationship was observed between the flow rate and particle size, Table 1 showed a reduction in particle size as the flow rate increased from 10 to 30 mL/min which could be expressed as increased flowing rate will increase the velocity of particles under the liquid nitrogen and decrease the opportunity of particles aggregation. Similar results were reported by Hu et al. (2003) for carbamazepine micronized particles prepared by SFCL technique.

### X-ray diffraction test of GP and GPNs

**Figure 2.**
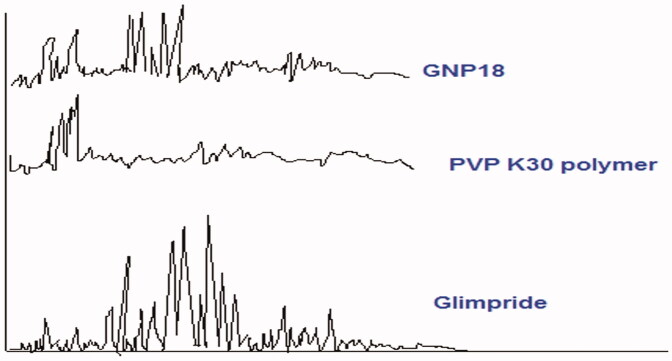
Powder X-ray diffraction patterns of Glimepiride, PVP K30, and GNP18 powders.

Figure 2 shows the X-ray diffraction patterns of GP, PVP K30 polymer, and GPN18 powders. Crystallinity was determined by comparing representative peak heights of GPN18 with the diffraction pattern of GP. The relative degree of crystallinity was calculated from the following equation (Calabrò et al., 2004):

(3) $$\text{The}\;\text{relative}\;\text{degree}\;\text{of}\;\text{crystallinity}\;=\;I_{\text{sample}}/I_{\text{reference}}$$

where: *I*_sample_ is the peak height of GPN18 and *I*_refrence_ is the peak height of GP at the same angle. The GP peak at 26.1° was used for calculating the relative degree of crystallinity of GPNs. The figure shows a reduction in the degree of crystallinity of the SCFL-nanoparticles of GP. This reduction in crystallinity is interpreted as a formation of a new solid with lower crystallinity. Furthermore, a reduced number of signals with lower intensity, showing the more amorphous features of GPNs compared with the drug molecules (Sharma et al., 2013).

**Figure 3.**
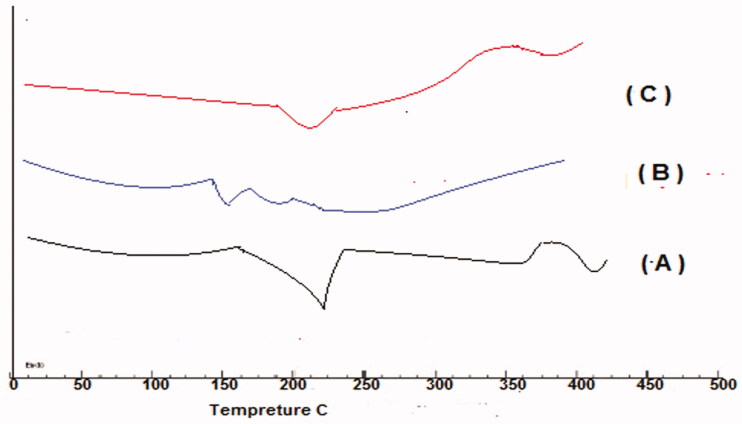
DSC of pure Glimepiride (A), DSC of PVP K30 polymer (B), DSC of GPN18 (C).

### Differential scanning calorimetry study

Thermograms of pure GP, PVP K30, and optimized GPNs formula (GPN18) are shown in Figure 3. The Thermogram of GP in a pure state showed a sharp endothermic peak at 229.57 °C which corresponds to the melting point of the polymorph I of the drug (Reginald-Opara et al., 2015). The transition temperature of the polymer ranged between 140 and 170 °C (B), thermogram of the optimized formula did not reveal any significant shift in the peak with a reduction in intensity compared to pure GP due to the decrease in drug amount.

### Analysis of water residuals and acetonitrile residual in GPNs

Three formulations prepared by different volumes of acetonitrile namely GPN9, GPN18, and GNP27 were tested for water residuals using KFT and the organic solvent residual levels by gas chromatography analysis. There was no water detected in the SCFL nano-sized powders. No organic solvents residuals were detected in any of the three formulations powders. The calibration standard was started from 25 ppm. The residual acetonitrile levels were less than 25 ppm in the powders which is lower than the allowed limit of the organic solvent determined by The International Conference on Harmonization (ICH) guidance. The guidance was classified acetonitrile as Class II solvents, which have a limit range from 100 to 1000 ppm in pharmaceutical formulations (U.S. Department of Health and Human Services, 1997).

### Measuring of the wettability of GPNs powders

**Figure 4.**
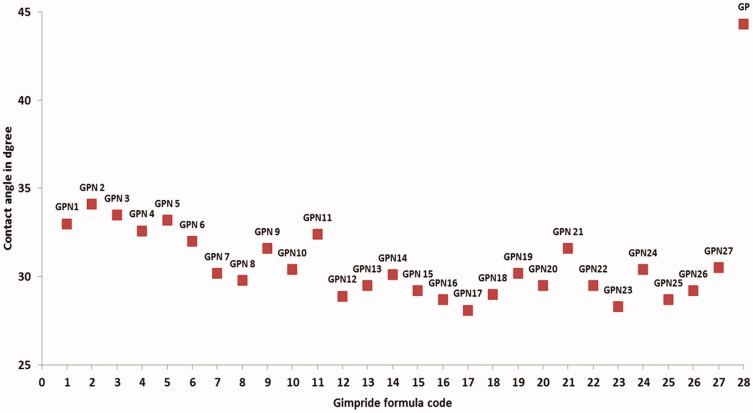
Contact angle of GPNS and pure glimepiride.

The contact angle test was used as an indicating method for the wettability of SCFL GP powders by purified water, which will be used as a dissolution media. The contact angles for the GPNs powders and pure GP powders are reported in Figure 4. The limits of contact angle are taken as reported in Martin (Martin, 1993), 0° for a complete wetting and 180° for no wetting. The value of contact angle was ranged between 28 ± 1.6° and 34 ± 1.5° for GNP2 and GPN18 respectively, which were significantly lower than that of GP powder 44 ± 3.2° at *p < .*05. The increased wettability of the GPNs powder compared to pure GP powders can mainly attribute to the amorphous state of the GP in GPNs particles, the reduction in the particle size, and the use of solubilizing polymer in the formulations (Chaudhari et al., 2012).

### Surface morphology

**Figure 5.**
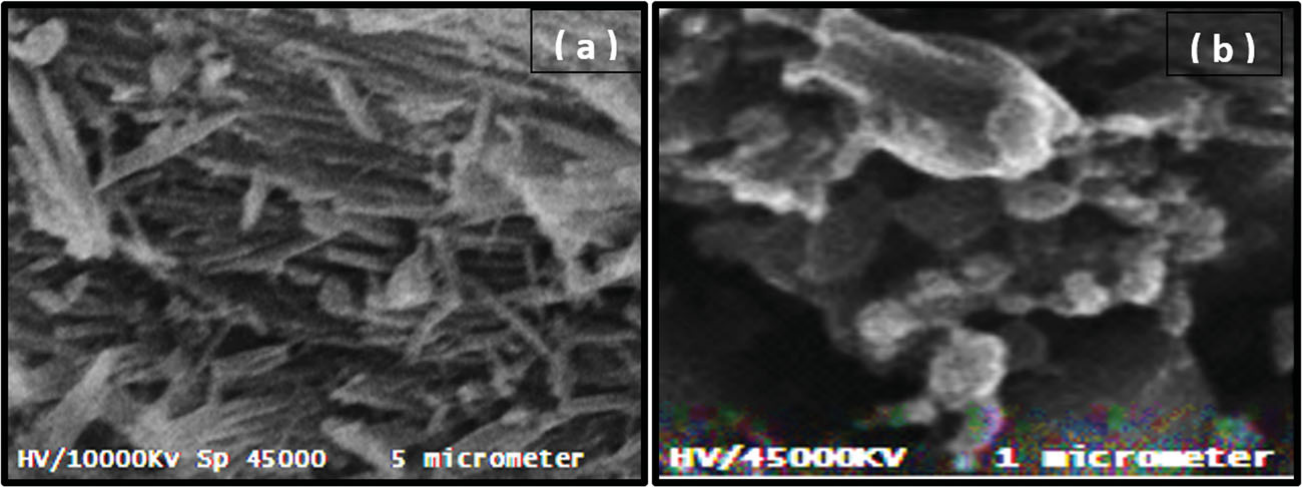
SEM micrographs of bulk GP (a), and selected formula of GPN particles (b).

SEM method was used to examine the surface morphology of GP nanosized particles produced by SCFL. The SEM micrograph of bulk GP (Figure 5(a)) showed large crystalline rhomboids with fractured sharp edges. In Figure 5(b), the SEM micrograph indicated the GPN particles had a spherical rough highly porous morphology with many small porous aggregates.

### Evaluation of GPNs saturation solubility in water

**Figure 6.**
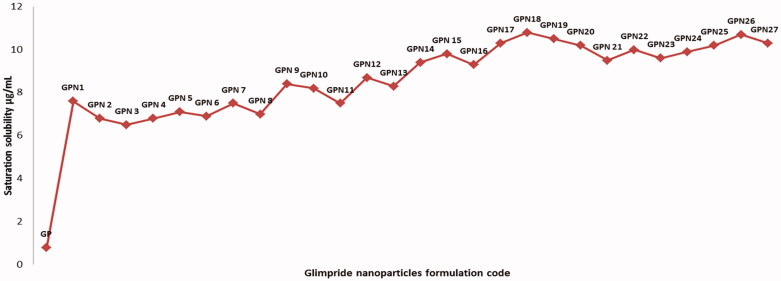
Saturation solubility of pure GP and GPNs formulations in water.

The saturation solubility of GPNs and pure GP was analyzed in distilled water. The solubility of GP from GPNs in water was enhanced significantly at *p* < .05 level compared with pure GP. The solubility of a pure drug in water was 0.8 ± 0.43 μg/mL, while the solubility of GP in GPNs formulation was varied between 6.5 ± 0.81 μg/mL and 10.8 ± 0.35 μg/mL in GPN3 and GPN18, respectively. The highest solubility enhancement was reported for GPN15; it showed 13.5 times enhancing in saturation solubility in comparison to pure GP as represented in Figure 6. This improvement in solubility could be interpreted based on the use of water-soluble Polyvinyl pyrrolidone derivatives which have a convenient ability to enhance the solubility of many poorly soluble drugs (Jijun et al., 2011). In addition, the amorphous state of GP in GPNs and the reduction in the particle size and consequent increase in its surface area led to a credible increase in GP solubility (Ghareeb et al., 2009).

### *In vitro* dissolution study

**Figure 7.**
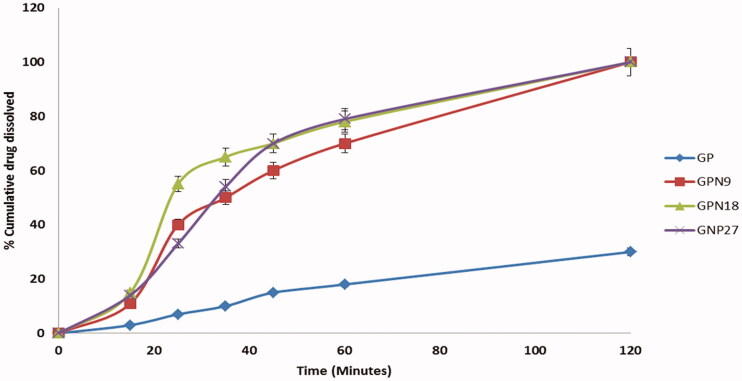
The cumulative percentages of drug dissolved versus time profiles of pure GP, GPN9, GPN18, and GPN27.

Based on particle size results, and saturation solubility data; GPNs formulations showed optimal results from different processing conditions were studied for *in vitro* dissolution profiles. Dissolution profiles of pure drugs, GNP9, GNP18, and GNP27 (*n* = 3) are presented in Figure 7. The rate of dissolution of pure GP was slow, while a significant enhancement in the dissolution rate of GP from nano-particles was observed. In 15 min, 11.5 ± 2.1%, 15.66 ± 4.1%, and 14.15 ± 2.5% of GP were released from GNP9, GNP18, and GNP27, respectively, compared to 3.42 ± 1.02% of pure GP. After 60 min only 18.83 ± 3.66% of GP was dissolved while 70.75 ± 5.4%, 78.3 ± 3.79%, and 79.04 ± 2.5% of GP were released from GNP9, GNP18, and GNP27, respectively. These results could be expressed based on the use of solubilizing agent (PVP K30); that many studies stated the enhancement of the dissolution of insoluble drugs by using solubilizing agents, such as soluble PVP grades, soluble form of chitosan, and so on (Mahapatra et al., 2019). The other factor that plays a major role in solubility enhancement is the nanosize, porous, and amorphous structure of GP in GPNs formulations; that give a large surface area available for dissolution in addition to the high internal energy of the amorphous state compared to the crystalline state that led to a faster dissolution rate. The reverse relationship between the dissolution rate and the particle size was verified by many studies, which deliberated the relation between the particle size and the solubility of poorly soluble drugs (Soni et al., 2017; Cai et al., 2018). At the same time, the greater wettability increased the dissolution rate of the SFCL nanosized powder (Chaudhari et al., 2012).

### Antidiabetic activity of selected formula of GNPs

**Figure 8.**
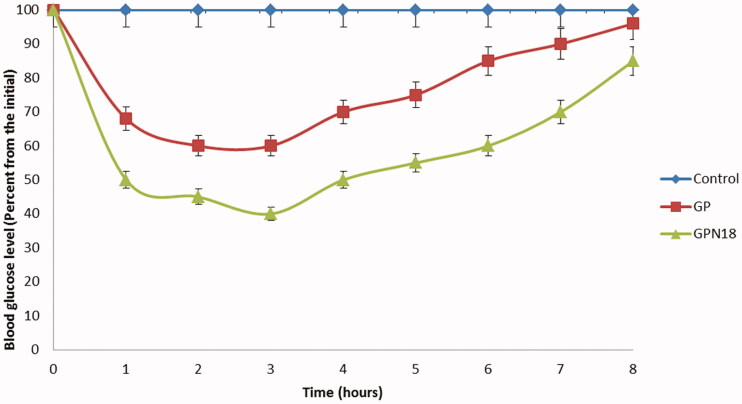
The antidiabetic activity of GP and GPN18 represented as a percentage of blood glucose from the initial versus time, mean ± SD.

GPN18 showed accepted results in particle size, solubility, and *in vitro* release studies for that, it was chosen for further investigation for antidiabetic activity and *in vivo* bioavailability study. The antidiabetic activity of GPN18 was evaluated in comparison with pure GP in streptozotocin-diabetic rats. Figure 8 showed that GPN18 has a higher and more rapid reduction in blood glucose levels in diabetic rats, which revealed the ability of the cryogenic technique to enhance the bioavailability of GP *in vivo*. Results showed that GPN18 reduce blood glucose levels to 50.2 ± 3.52% of the initial value after 1 h, while the pure GP showed a reduction in glucose levels to 32.3 ± 4.61% of the initial value. 72.3 ± 3.42% and 55.1 ± 2.68% reduction of the initial values in blood glucose levels was observed after 4 h. These results may be expressed based on enhancing the solubility of GP in GNPs and the use of a hydrophilic carrier (PVP K30) and surfactant. Similar results were reported by Qushawy et al. (2020) and Li et al. (2016) who prepared GP solid dispersion and microemulsion for improving its solubility and hence bioavailability.

### In vivo bioavailability study

**Figure 9.**
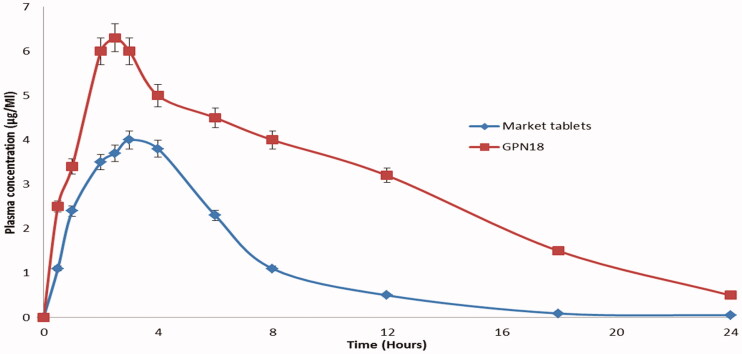
Plasma concentration time profiles pf GP after oral administrations of GPN18 and commercial tablets in rats (mean ± SD).

Plasma levels of GP after oral administration of GPN18 and commercial tablets showed that SCFL nanoparticles enhanced the absorption of GP compared with the control tablets. Table 2 and Figure 9 showed that the maximum plasma concentrations of GP were 6.30 ± 1.2 and 4.01 ± 0.85 μg/mL for GPN18 and commercial tablets respectively. The enhancement of *C*_max_ was 1.58-fold for GPN18 as compared with the commercial one. Also, there was a significant increase in the area under the plasma concentration–time curve of GP after oral administration of GPN18 (35.67 ± 4.30 μg/mL h) and control tablets (19.82 ± 3.0 μg/mL h). The time of maximum concentration was 2.58 ± 0.12 h for GPN18 while it was 2.91 ± 0.07 h for commercial tablets, this difference in *T*_max_ could be due to the increase in the solubility of GP in GPN18 compared with the control tablets. The study showed an enhancement in the bioavailability (1.79-folds) of GP after oral administration of SCFL nanoparticles. Results could be understood based on the increase in the solubility and the decrease in the particle size and hence the increase in the surface area and absorption of GP (Ganesan et al., 2015). Nanocrystals of many drugs reported a significant enhancement in solubility, dissolution, and eventually a more rapid rate of absorption compared with its commercial conventional dosage forms (Shah et al., 2016; Haroon et al., 2017).

**Table 2.** Measured pharmacokinetics of GP following an oral administration of GPN18 and marketed tablets in rats.

Pharmacokinetic parameter	GPN18	Marketed tablets
*C*_max_, µg/mL	6.30 ± 1.2	4.01 ± 0.85
*T*_max_, h	2.58 ± 0.12	2.91 ± 0.07
AUC_0–24_, µg/L^ ^h	35.67 ± 4.30	19.82 ± 3.0 μg/mL h
MRT, h	9.6	8.3
Rel F (%)	179.97	–

## Conclusions

The spray freezing into cryogenic liquids technique was found to be useful in enhancing the dissolution rate of GP. The processing variabilities including the ratio of drug to carrier, feeding solution volume, and feed flow rate played a significant role in improving the dissolution rate and hence the hypoglycemic effect of GP *in vivo*. The study revealed that all formulations have accepted high production yield and drug content. The results concluded that the drug-carrier ratio 1:2, in a 150 mL feeding solution pumped at a flow rate of 30 mL/min showed a noticeable enhancement in GP dissolution rate and a 1.79-fold increase in its bioavailability. In conclusion, the SCFL technique using an acetonitrile system is an effective particle reduction process for improving the dissolution rates of poorly water-soluble drugs.
